# T Cells Contain an RNase-Insensitive Inhibitor of APOBEC3G Deaminase Activity

**DOI:** 10.1371/journal.ppat.0030135

**Published:** 2007-09-21

**Authors:** Beth K Thielen, Kevin C Klein, Lorne W Walker, Mary Rieck, Jane H Buckner, Garrett W Tomblingson, Jaisri R Lingappa

**Affiliations:** 1 Department of Pathobiology, University of Washington, Seattle, Washington, United States of America; 2 Translational Research Program, Benaroya Research Institute at Virginia Mason, Seattle, Washington, United States of America; 3 Department of Medicine, University of Washington, Seattle, Washington, United States of America; King's College London, United Kingdom

## Abstract

The deoxycytidine deaminase APOBEC3G (A3G) is expressed in human T cells and inhibits HIV-1 replication. When transfected into A3G-deficient epithelial cell lines, A3G induces catastrophic hypermutation by deaminating the HIV-1 genome. Interestingly, studies suggest that endogenous A3G in T cells induces less hypermutation than would be expected. However, to date, the specific deaminase activity of endogenous A3G in human CD4+ T cells has not been examined directly. Here, we compared deaminase activity of endogenous and exogenous A3G in various human cell lines using a standard assay and a novel, quantitative, high-throughput assay. Exogenous A3G in epithelial cell lysates displayed deaminase activity only following RNase treatment, as expected given that A3G is known to form an enzymatically inactive RNA-containing complex. Surprisingly, comparable amounts of endogenous A3G from T cell lines or from resting or activated primary CD4+ T cells exhibited minimal deaminase activity, despite RNase treatment. Specific deaminase activity of endogenous A3G in H9, CEM, and other T cell lines was up to 36-fold lower than specific activity of exogenous A3G in epithelial-derived cell lines. Furthermore, RNase-treated T cell lysates conferred a dose-dependent inhibition to epithelial cell lysates expressing enzymatically active A3G. These studies suggest that T cells, unlike epithelial-derived cell lines, express an unidentified RNase-resistant factor that inhibits A3G deaminase activity. This factor could be responsible for reduced levels of hypermutation in T cells, and its identification and blockade could offer a means for increasing antiretroviral intrinsic immunity of T cells.

## Author Summary

APOBEC3G (A3G) is an antiviral enzyme that is expressed in human T cells and macrophages, which are the cell types infected by HIV. Early in the HIV life cycle, the HIV RNA genome is reverse transcribed into DNA. A3G can modify this DNA enzymatically, leading to high rates of mutation such that the virus can no longer replicate. To date, most studies of A3G's enzymatic activity have utilized cell lines (293T and HeLa) that can be transfected to express A3G but do not express it endogenously. A report of unexpectedly low levels of mutation in viral DNA from HIV-infected human T cells led us to investigate regulation of A3G enzymatic activity in T cells. We developed a high-throughput assay to compare the enzymatic activity of endogenous A3G in T cells versus transfected (exogenous) A3G. Surprisingly, enzymatic activity of A3G from human T cell lines and primary T cells was very low relative to A3G from transfected cells, even when corrected for A3G protein amount. Moreover, T cell lysates inhibited enzymatic activity of exogenously expressed A3G. These data suggest that enzymatic activity of endogenous A3G in human T cells is inhibited by an uncharacterized mechanism that may protect the host from this DNA mutator and could have important implications for A3G antiviral activity in vivo.

## Introduction

Viral infection represents a common threat faced by most cells, and different cell types have evolved unique strategies for defending against viral pathogens. One such strategy involves the deoxycytidine deaminase APOBEC3G (A3G), an intrinsic defense mechanism specific to primates. A3G is a cellular protein expressed in a limited number of cell types [1,2], including, but not limited to, T cells and macrophages, and has antiviral activity against HIV-1, hepatitis B virus, and endogenous retroelements (reviewed in [3]). During HIV-1 infection, A3G can exert antiviral effects either when it is packaged into virions (reviewed in [4]) or when it is present in T cells [5], which are a natural target of infection. The antiviral effect of packaged A3G is not observed in infections with wild-type virus because HIV encodes the viral infectivity factor (Vif), which prevents A3G from being packaged into newly formed virus particles by targeting it for proteosomal degradation [6–9] and by other mechanisms [10]. However, in the absence of functional Vif, A3G is packaged and subsequently mediates deamination of deoxycytidine (dC) residues in the nascent minus-strand DNA during reverse transcription of the HIV genome. As a result of this deamination, G-to-A hypermutation of the coding strand can occur, resulting in an increased proportion of non-infectious virus (reviewed in [4]). Alternatively, the presence of multiple deoxyuridines (dUs) in the minus strand may prevent accumulation of reverse transcripts, either by triggering degradation by cellular DNA repair machinery [11–13] or by impairing synthesis [14,15]. In both cases, dC-to-dU deamination was initially thought to be critical to the antiviral effects of packaged A3G. However, subsequent studies have demonstrated that packaged A3G mutants can have antiviral effects even when they lack deaminase activity [16–19].

Recent studies also indicate that A3G does not always need to be packaged to inhibit HIV infection. Endogenous A3G present in resting CD4+ T cells from peripheral blood, but not from lymphoid tissue, restricts infection of these cells in a Vif-independent manner [5,20]. Interestingly, when reverse transcripts from these cells were examined, unexpectedly low levels of hypermutation were observed [5], raising the possibility that A3G-mediated restriction of infection of resting T cells may also occur by mechanisms other than deamination. Thus, both packaged A3G and A3G present in resting T cells may restrict HIV-1 infection by a deaminase-independent mechanism, although in both cases some degree of mutation of HIV reverse transcripts is typically seen.

To determine how much deamination contributes to the antiviral effect of A3G in vivo, a better understanding of the deaminase activity of endogenous A3G in T cells and macrophages is needed. Regulation of A3G's deaminase activity has been studied primarily using tagged A3G expressed exogenously in epithelial cell lines. In these cells, exogenously expressed A3G is found in high molecular mass (HMM) ribonucleoprotein complexes, which are enzymatically inactive. RNase A treatment of epithelial cell lysates disrupts the integrity of these complexes, producing an enzymatically active, low molecular mass (LMM) form of A3G [5,20,21]. RNase-sensitive HMM complexes are also found in T cell lines, activated CD4+ T cells, resting CD4+ T cells from lymphoid tissue, and CD16+ monocytes, all of which are susceptible to infection by wild-type HIV-1. Interestingly, resting peripheral blood CD4+ T cells and CD16- monocytes, which restrict HIV-1 infection, contain primarily LMM A3G [5,20–22]. However, to date, no studies have examined enzymatic activity of endogenous A3G in resting or activated CD4+ T cells. Although the finding that LMM A3G in resting T cells confers resistance to HIV infection demonstrates its antiviral activity, the surprisingly low percentage of mutated HIV reverse transcripts recovered from these cells [5] raises the possibility that A3G may not be as enzymatically active in resting T cells as it is in epithelial cell lines. Here, we demonstrate that the deaminase activity of LMM A3G in T cells is much lower than the deaminase activity of comparable amounts of exogenous A3G expressed in epithelial cells. These data suggest that endogenous A3G in T cells is regulated by a novel inhibitory protein or proteins. This inhibition of A3G deaminase activity could explain the unexpectedly low level of HIV hypermutation observed in resting T cells [5]. Such a mechanism for regulation in cells expressing endogenous A3G in the LMM form could allow for more fine-tuned modulation of this enzyme that may be harmful to the cell if uncontrolled but is also needed for defense against viruses and endogenous retroelements [23].

## Results

**Figure 1:**
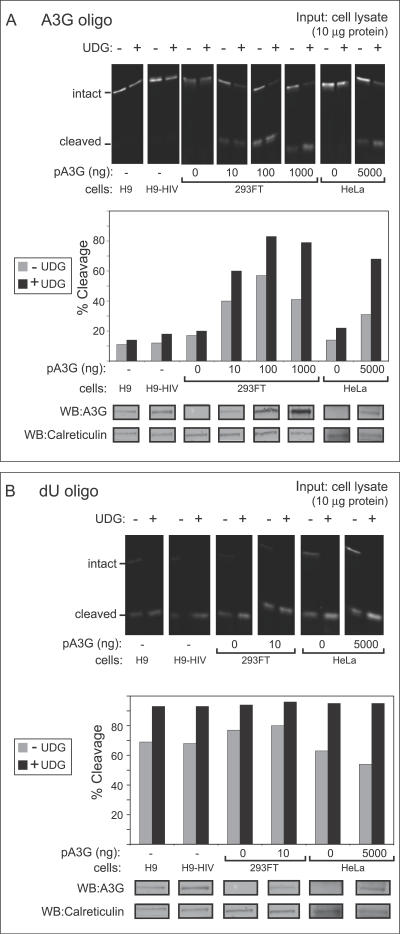
A Gel-Based Assay Reveals That Endogenous A3G in T Cell Lines Exhibits Unexpectedly Low Deaminase Activity Compared to Exogenous A3G in Transfected Epithelial-Derived Cell Lines (A) Deaminase activity was measured using an infrared 700 (IR700)–labeled oligo containing the A3G recognition site (CCC) either with or without exogenous recombinant uracil DNA glycosylase (+/- UDG). Oligos were incubated with crude cell lysates containing 10 μg of total cellular protein obtained from H9 cells, H9 cells expressing the HIV genome containing a deletion in Vif (H9-HIV), or from HeLa or 293FT cells transfected with the indicated amounts of A3G plasmid DNA (pA3G). Extent of oligo cleavage (indicating extent of deamination) was determined by gel electrophoresis followed by detection on a LI-COR scanner (top panel), and the percentage of probe cleaved was graphed (second panel). Below, equivalent amounts of cell lysate were analyzed in parallel by western blot (WB) to show A3G protein content. Western blot of calreticulin is shown as a loading control. (B) UDG activity was measured in select lysates from (A) using an IR700-labeled dU-containing oligo in the presence or absence of exogenous UDG (+/- UDG). Results are displayed as in (A) and show that unlike A3G activity shown in (A), UDG activity is similar in all cell lysates analyzed. All assays were performed on RNAse A–treated samples.

To determine if A3G activity is regulated differently in T cells and epithelial cells, we compared the activity of endogenous A3G in human T cell lines (H9 cells and H9 cells expressing the HIV genome without Vif) to the activity of comparable amounts of exogenous A3G expressed in human epithelial-derived cell lines (293FT and HeLa cells) using a standard gel-based deaminase assay, in which an infrared 700 (IRDye 700)–labeled oligonucleotide containing the A3G deamination motif (dCdC) was incubated with samples of total cell extracts containing 10 μg of total cellular protein. Uracil DNA glycosylases (UDGs) present endogenously in cell extracts and recombinant UDG added to the assay convert deaminated dCs to abasic sites, which are cleaved during the assay upon incubation at a basic pH (see Figure S1A). The resulting oligonucleotide cleavage is then detected by gel electrophoresis followed by imaging with a LI-COR infrared scanner. While incubation of the oligonucleotide with RNase A–treated epithelial cell extracts expressing A3G resulted in high levels of cleavage in the absence or presence of exogenous UDG, incubation with RNase A–treated H9 cell extracts resulted in much less oligonucleotide cleavage, even in the presence of exogenous UDG (Figure 1A). An aliquot of each cell lysate subjected to immunoblotting for A3G confirmed the presence of similar amounts of A3G in lysates of H9 cells and transfected 293FT (Figure 1A). The surprising finding that endogenous A3G in the T cell extracts exhibited less deaminase activity than comparable amounts of transfected A3G in epithelial cells raised the possibility that an inhibitory activity exists in H9 T cells. Since two enzymatic reactions occur in this assay (deamination by A3G and base excision by UDG), a parallel control was performed to determine which of the two reactions was inhibited in H9 cells. When an IR-labeled oligonucleotide containing dU was incubated with the same cell extracts in the presence of RNase A but without exogenous UDG, it was cleaved to a similar extent across cells types (Figure 1B), indicating equivalent endogenous UDG-mediated base excision in H9 T cell lines and epithelial cell lines. These data are consistent with previous reports [24] and indicate that H9 extracts do not contain an inhibitor of UDG activity. Together, these findings suggested that after RNase A treatment, endogenous A3G in H9 T cells exhibits less A3G deaminase activity than comparable amounts of transfected A3G expressed in 293FT cells. Since improved and nearly complete cleavage was observed in all cell types upon supplementation with exogenous UDG, all future studies were performed in the presence of exogenous UDG.

To quantify A3G activity accurately, it would be important to use an assay that yields activity measurements proportional to the amount of A3G input over a wide range of enzyme amounts. The time- and labor-intensive gel-based assay is not readily amenable to the multiple measurements that are required to validate linearity of the assay for a large number of cell types. Thus, to obtain a more quantitative view of A3G deaminase activity in different cell types, we developed a fluorescence resonance energy transfer (FRET)–based, high-throughput assay that would allow us to more easily perform dose-response curves and multiple replicates using either crude cell extracts or immunoprecipitated A3G. The lack of simple, non-radioactive, quantitative assays has been at least partially responsible for the absence of comprehensive studies of A3G activity in a wide variety of cell types and physiological situations. Although many studies examine G-to-A mutation rates by either E. coli–based mutation assays [17,18,25,26] or sequencing the HIV provirus [5,16,27,28], such assays reflect A3G deaminase activity only indirectly. One method for directly assaying A3G deaminase activity measures the ability of immunoprecipitated or virion-incorporated A3G to induce deamination-dependent cleavage of ^32^P-labeled oligonucleotides, as detected by altered migration on a gel [5,26,29]. Alternatively, incorporation of ^3^H-labeled dATP, which only occurs when dCs in an oligonucleotide template strand are converted to dUs by deamination, can be measured [30,31]. We devised a non-radioactive assay in which the A3G substrate is a 13-base oligonucleotide that contains the A3G recognition motif and is dual-labeled with FRET-optimized fluorophores. We added exogenous UDG to ensure complete uracil excision. Treatment with base leads to cleavage of the labeled oligonucleotide at sites of deamination, as in the gel-based assay. Upon cleavage, the reporter fluorophore is no longer efficiently quenched, producing an increase in detectable fluorescence (Figure S1A). Using different ratios of double- and single-labeled oligos (Table S1), we demonstrated that fluorescence is directly proportional to the amount of single-labeled oligo representing cleaved substrate (Figure S1B).

We then validated the FRET-based assay by demonstrating that increasing A3G expression in 293FT cells resulted in a dose-dependent increase in deaminase activity when the assay was performed with extracts from cells expressing increasing amounts of A3G plasmid, but only in the presence of RNase A (Figure S2A). These data are consistent with previous reports that A3G in 293T cells is only active as a deaminase after conversion from the HMM to LMM form using RNase A [5]. Dose dependence was also observed when the deaminase assay was performed with increasing amounts of cell lysate from 293FT cells transfected with a fixed amount of A3G plasmid and when the length of oligonucleotides containing the A3G cleavage site was varied (Figure S2B). The consistent low level of cleavage that was detected in cell lysates lacking significant A3G expression (Figures S2A and S2B) is likely due, at least in part, to other cytidine deaminase enzymes, given that the degree of cleavage induced by incubation with crude cell lysates lacking A3G was greater for CCC-containing oligos than for GGG-containing oligos (Figure S2A). While specific antibodies are not available for most APOBEC3 family members, we were able to assess A3F levels using an antibody that specifically recognizes A3F and not A3G (Figure S2C). In some experiments, we detected expression of APOBEC3F (A3F) in non-transfected 293FT cells, albeit at low levels (unpublished data). This may account for the A3G-independent baseline activity we observed in these cells since some of our oligos (shown in Table S1) also contain the TC dinucleotide motif recognized by A3F [32]. Nevertheless, we consistently observed activity above background levels that was mediated by A3G in a dose-dependent manner and encompassed a dynamic range of 900–1,100 relative fluorescence units (RFU). Additionally, although others have reported a minimum length requirement of 16 bases for oligo binding by purified recombinant A3G [33], we were able to detect deamination by A3G from cell extracts using the FRET-based assay with oligos of 13 to 21 bases in length (Figure S2B; unpublished data).

To measure the activity of A3G in the absence of cellular nucleases and other deaminases, we subjected lysates from 293FT cells transfected with A3G to immunoprecipitation with beads coupled to either affinity-purified A3G-specific antiserum (αA3G) or rabbit IgG under native conditions before (Figure S2D) or after (unpublished data) RNase A treatment. Using either approach, enzymatically active A3G was efficiently recovered following immunoprecipitation with αA3G, and increasing amounts of immunoprecipitation eluate resulted in a dose-dependent increase in A3G activity spanning a range of 833 RFU. Note that the A3G antiserum most likely immunoprecipitates A3G specifically since it is directed against the C-terminus of A3G, which differs considerably from the C-termini of other APOBEC family members. Consistent with this, A3G immunoprecipitation completely eliminates the baseline A3G-independent deamination activity that is observed when activity is measured in crude cell lysates (compare Figure S2D with Figure S2A and S2B). Finally, to further validate the FRET-based assay, we demonstrated that two A3G mutants that contain substitutions in both deaminase domains and are known to display substantially reduced enzymatic activity in E. coli–based mutation assays [17] also displayed 30- to 50-fold less activity than wild-type A3G in the FRET-based assay (Figure S2E). Thus, taken together, these results demonstrate that the FRET-based assay specifically detects catalytically active, exogenously expressed A3G from both crude cell lysates and immunoprecipitations over a dynamic range of 800 to 1,100 RFU.

**Figure 2:**
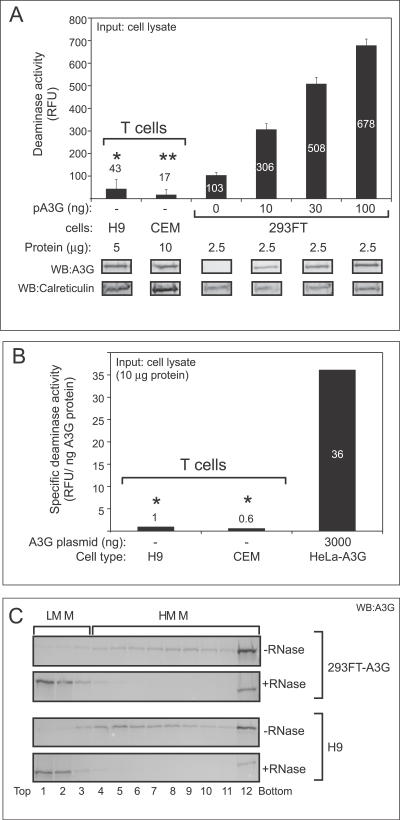
A Quantitative FRET-Based Assay Reveals That Endogenous A3G in T Cell Lines Exhibits Very Low Specific Deaminase Activity Compared to Transfected A3G Expressed in Epithelial-Derived Cell Lines (A) Deaminase activity of crude cell lysates from H9 and CEM T cell lines or from 293FT cells transfected with the indicated amounts of A3G plasmid DNA (pA3G) was determined as RFU using the FRET-based assay and was graphed. Variable amounts of total cellular protein inputs were used as indicated (μg protein) to match the total amount of endogenous A3G from T cell lines with the highest level of transfected A3G in 293FT cells of epithelial origin. Below, western blots (WB) show amount of A3G input for all samples, as well as amount of calreticulin as a reference for amount of total protein input. Statistical significance *, *p* < 0.01 relative to 293FT, 100 ng; **, *p* < 0.01 relative to 293FT, 30 ng. Background fluorescence obtained for each sample using the 13-mer GGG-containing oligonucleotide was subtracted from the signal detected using the 13-mer CCC-containing substrate. Error bars represent standard error of the mean of triplicate reactions. Note that when the A3G dose-response deaminase activity values for each 293FT sample were graphed relative to amounts of A3G protein in those samples determined by densitometry, a linear relationship with a correlation coefficient of 0.99 was observed (unpublished data). (B) Specific A3G deaminase activity (RFU/ng A3G protein) of A3G expressed endogenously in T cells or expressed exogenously by transfection in HeLa cells was calculated from a typical experiment, as shown in Figure S3, and was graphed. Statistical significance *, *p* < 0.05 relative to HeLa control. (C) Cell lysates from A3G expressing 293FT and H9 cells +/- RNase A treatment were subjected to velocity sedimentation, fractionated (1–12), and analyzed by western blotting with antibody to A3G. Western blots show that RNase treatment was effective in converting HMM A3G to LMM A3G in all cell types analyzed. Brackets indicate migration of A3G HMM and LMM complexes. All panels are representative of at least three independent experiments. All FRET assays were performed with RNase A. Numbers on/above bars in (A and B) indicate actual values graphed.

Using our quantitative FRET-based assay for A3G activity, we asked whether A3G expressed endogenously in human cell lines exhibits similar levels of deaminase activity as A3G transfected into epithelial cells. For this purpose we used two human T cell lines: the H9 cells, described above, as well as CEM cells, which also express native A3G, albeit at lower levels than H9 cells. Moreover, for each T cell line, we chose input cell lysate amounts that were matched for A3G content with samples from transfected 293FT cells displaying high deaminase activity (i.e., transfected with 30–100 ng of A3G plasmid). As expected, transfected A3G in 293FT cell lysate exhibited a dose-dependent increase in deaminase activity that was proportional to the relative amounts of A3G protein expressed by transfecting increasing amounts of plasmid DNA (Figure 2A). However, the signal produced by RNase-treated H9 cell lysate was 15-fold less than the signal produced by the lysate of 293FT cells containing a comparable amount of transfected A3G that was treated with RNase A and assayed in parallel (Figure 2A; 43 RFU versus 678 RFU), consistent with what was found with the gel-based assay (Figure 1A). Similarly, RNase-treated CEM cell lysate produced a signal that was 30-fold less than a comparable amount of A3G expressed by transfection of 293FT cells (Figure 2A; 17 RFU versus 508 RFU). As expected, the amount of input cell lysate needed to achieve these levels of A3G was higher for CEM cells (as indicated by higher levels of the calreticulin loading control) due to their relatively low level of A3G expression [34].

To compare deaminase activity in different cell types, we determined the specific activity of A3G in each cell type. We first determined the amount of A3G expressed at steady state in T cell lines and transfected HeLa cells by interpolation on a standard curve generated by parallel immunoblotting of aliquots of samples and known amounts of recombinant standards (Figure S3A). H9 and CEM cells contained approximately 2.9 and 2.0 ng A3G/μg cellular protein, respectively, which was similar to the amount of A3G expressed in the transfected HeLa control (Figure S3). We then calculated specific deaminase activity (deaminase activity per unit A3G protein) by normalizing the deaminase activity of each sample to the amount of A3G protein in each sample (Figure S3B), and found that the specific deaminase activity of endogenously expressed A3G in T cell lines was 36-fold lower than that of A3G transfected into HeLa cells (Figure 2B). Thus, even when normalized for A3G protein levels, deaminase activity appears to be inhibited in T cells.

Previous work showed that RNase treatment was needed to dissociate HMM complexes, which in turn was required for A3G enzymatic activity [5]. While the concentration of RNase A we used in the experiments reported here (250 μg/ml) was 5-fold greater than that used by others to dissociate HMM complexes [5] and 250-fold above that needed to reduce cellular RNA levels by 1,000-fold [35], we wanted to verify that RNase treatment was indeed disrupting HMM complexes in T cells, given their lower level of deaminase activity. Velocity sedimentation gradients demonstrated that RNase A converted A3G in T cell lines from an HMM form to an LMM form (Figure 2C), indicating the effectiveness of the RNase A treatment. Thus, the low A3G activity in T cells is not due to A3G being retained in the HMM complex.

**Figure 3:**
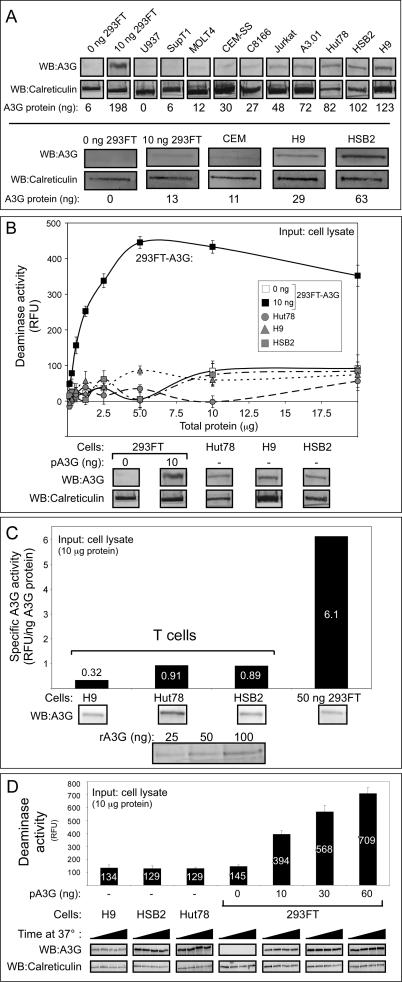
Other T Cell Lines with High Levels of Endogenous A3G Protein Exhibit Very Low Specific Deaminase Activity (A) Endogenous A3G protein levels from the indicated T cell lines, compared to 293FT cells mock-transfected (0 ng 293FT) or transfected with 10 ng A3G plasmid DNA, were determined by western blotting (WB) in two independent experiments (top versus bottom panels). Western blots of calreticulin are shown as a loading control. Amount of A3G protein (ng) is indicated in each lane, as determined by densitometry relative to recombinant A3G protein standards that are not shown. (B) Deaminase activity of different amounts RNase A–treated crude cell lysates either from A3G-expressing T cells or from 293FT cells transfected with 0 or 10 ng of A3G plasmid DNA (pA3G) was determined using the FRET-based assay and was graphed. Amounts of input cell lysate are indicated by μg of total cellular protein. Error bars represent standard error of the mean of triplicate reactions. Below, western blots show relative amounts of A3G compared to the loading control calreticulin for each cell type. (C) Specific deaminase activity of A3G (RFU/ng A3G protein) was calculated for DNase-treated lysates of H9, HSB2, and Hut78 cells, as well as for 293FT cells transfected with 50 ng A3G plasmid DNA (50 ng 293FT) from a representative experiment and were graphed. Below, western blots show the amount of A3G in each cell lysate and in known recombinant standards (rA3G), which were used to determine protein amounts for specific deaminase activity calculations, as described in Figure S3. Numbers on/above bars indicate actual values graphed. (D) Western blots show the amount of A3G and calreticulin in equivalent amounts of crude cell lysates from H9, HSB2, and Hut78 T cells or from 293FT cells transfected with the indicated amounts of A3G plasmid DNA (pA3G) and incubated for 0, 30, 60, and 90 min at 37 °C (increased time from left to right). Corresponding A3G activity was determined for all time points using the FRET-based assay, and the maximal activity (90 min) is graphed above. Background fluorescence for each sample using the 13-mer GGG-containing oligonucleotide was subtracted from signal detected with the 13-mer CCC-containing substrate for all FRET deaminase assays. Numbers on/above bars indicate actual values graphed. All panels are representative of three independent experiments. All FRET assays were performed with RNase A.

Since different T cell lines express significantly different amounts of A3G, we examined expression of A3G in several other T cell and monocyte lines by immunoblotting. Although many expressed little or no A3G (SupT1, U937, MOLT4, CEMSS, C8166) and others expressed low levels of A3G (CEM, Jurkat, A3.01), two cell lines (HSB2 and Hut 78) displayed levels of A3G similar to that of H9 cells (Figure 3A). Notably, the relative amounts of A3G protein seen by immunoblotting correlated well with the relative amounts of A3G mRNA observed by others in these cell lines [34,36]. We examined the amount of deaminase activity in cell lines whose A3G amounts were within 2-fold of the amount of A3G observed in transfected 293FT cells. Despite excellent endogenous A3G expression, HSB2 and Hut78 cells, like H9 T cells, exhibited very little deaminase activity, even when assayed over a 20-fold range of total protein input (Figure 3B). Note that transfected A3G expressed in 293FT cells displays a linear increase in activity over approximately a 10-fold range of input amounts, with activity then reaching a plateau consistent with saturation of the assay. When activity was normalized for A3G protein levels, we found that A3G from 293FTs had a specific deaminase activity of 6.1 RFU/ng A3G, while specific deaminase activity for A3G from all T cell lines tested was significantly lower, ranging from 0.32 to 0.91 RFU/ng A3G (Figure 3C). Similar results were observed in lysates prepared in the presence (unpublished data) or absence (Figure 3C) of EDTA, and with (Figure 3C) or without (unpublished data) DNase treatment. Moreover, because the assay involves incubation of lysates for over an hour at 37 °C, we considered the possibility that degradation of A3G could occur over the course of the assay despite the use of a protease inhibitor cocktail during cell harvest, and that this could be more prominent in T cells than in other cell types. However, when aliquots of the assay were removed at the start (0 min) and during the assay (30, 60, and 90 min), no change was seen in the amount of A3G or calreticulin during the 37 °C incubation in H9, HSB2, Hut78, or transfected 293FT cell lines (Figure 3D), ruling out selective A3G degradation as a cause of the very low deaminase activity levels in T cell lines. Thus, several different T cell lines that express significant amounts of A3G exhibited very low specific activity compared to exogenous A3G expressed in 293FT cell lines, and these differences could not be explained by assay conditions or other experimental parameters.

**Figure 4:**
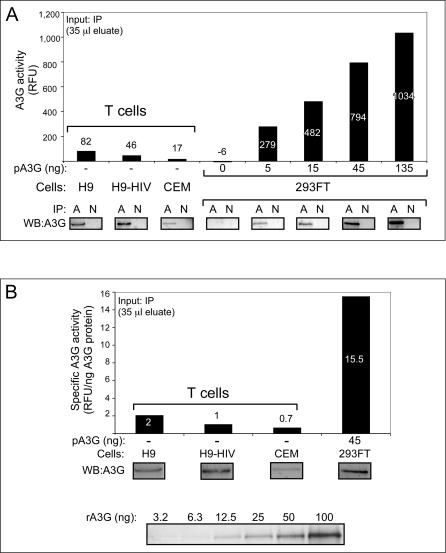
Endogenous A3G Immunoprecipitated from T Cell Lines Exhibits Very Low Specific Deaminase Activity Compared to Exogenous A3G Immunoprecipitated from Transfected Epithelial-Derived Cell Lines (A) Parallel IPs were performed using either specific A3G antisera (A) or a rabbit non-immune control (N) for H9 T cells, H9 T cells expressing the HIV genome containing a deletion in Vif (H9-HIV), CEM T cells, or 293FT cells transfected with the indicated amounts of A3G plasmid DNA (pA3G). Parallel aliquots of immunoprecipitation eluates were used to determine A3G deaminase activity using the FRET assay (35 μl; graph) and for western blots (10 μl; WB). FRET signal obtained from non-immune IPs was subtracted from signal obtained from the A3G IPs for each sample. (B) Specific deaminase activity of A3G (RFU/ng A3G protein) was determined for the indicated samples from (A). Below, western blots show amount of A3G in each immunoprecipitation and in known recombinant standards (rA3G), which were used to determine protein amounts for specific deaminase activity calculations. All panels are representative of at least three independent experiments. All FRET assays were performed with RNase A. Numbers on/above bars indicate actual values graphed.

To confirm that A3G or A3G-containing complexes are responsible for the findings obtained with crude cell lysates, we isolated A3G and associated proteins from other cellular components by immunoprecipitation with an antibody specific to A3G followed by elution under native conditions. To dissociate HMM complexes, immunoprecipitates were treated with RNase A. Again, despite RNase A treatment and the presence of similar amounts of A3G protein in the immunoprecipitation eluates, A3G immunoprecipitated from H9 and CEM cells displayed significantly less deaminase activity than A3G from 293FT cells (Figure 4A), suggesting that LMM A3G from T cells is less active than LMM A3G from transfected epithelial cell lines. Similar results were seen when lysates were pre-treated with RNase A prior to immunoprecipitation and when activity assays were performed directly on immunoprecipitation beads (unpublished data). No activity above baseline was seen in the corresponding H9 or CEM crude cell lysates or in immunoprecipitations that were not subjected to RNase treatment (unpublished data). SupT1, a T cell line that does not express A3G mRNA [34,36], lacked A3G protein by immunoblotting and did not display deaminase activity in our assay, as expected (unpublished data). Although the amount of A3G protein in the eluates from T cells was very similar to that of control 293FT cells, the specific activity of endogenous A3G immunoprecipitated from T cell lines was 7.8- to 22-fold lower than that of A3G transfected into 293FT cells (Figure 4B). Notably, little difference in A3G-specific activity was observed when A3G was immunoprecipitated from RNase A–treated lysates of parental H9 T cells versus H9 T cells stably expressing a Vif-deleted HIV-1 genomic construct (Figure 4B), indicating that expression of the HIV-1 provirus did not alter the surprisingly low activity of A3G immunoprecipitated from H9 cells.

**Figure 5:**
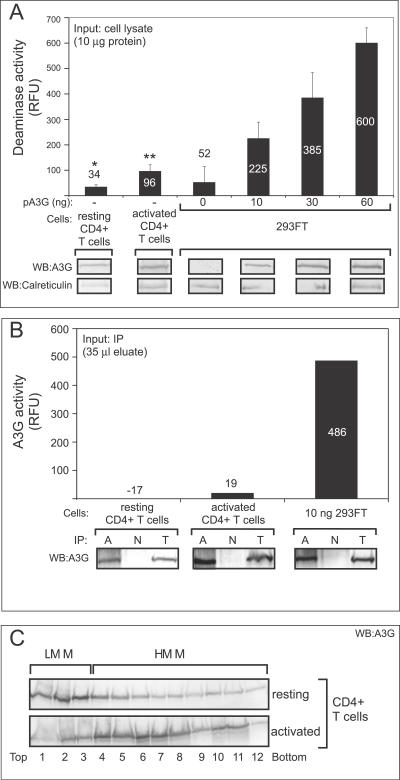
Endogenous A3G from Primary Human CD4+ T Cells Exhibits Very Low Specific Deaminase Activity (A) Deaminase activity of RNase A–treated crude cell lysates from primary CD4+ T cells, either resting or activated for 72 h, or from 293FT cells expressing the indicated amounts of A3G plasmid DNA (pA3G) was determined using the FRET-based assay and is graphed. Below, western blots (WB) show amount of A3G and the loading control calreticulin in equivalent aliquots of cell lysates. Background fluorescence obtained using the 13-mer GGG-containing oligonucleotide was subtracted from the signal detected using the 13-mer CCC-containing substrate. Error bars represent standard error of the mean of triplicate reactions. Statistical significance *, *p* < 0.01 relative to 293FT, 10 ng; **, *p* < 0.05 relative to 293FT, 30 ng. Numbers on/above bars indicate actual values graphed. (B) Parallel IPs were performed using either specific A3G antisera (A) or a rabbit non-immune control (N) for resting or activated CD4+ T cells or 293FT cells transfected with 10 ng A3G plasmid DNA (10 ng 293FT). Parallel aliquots of immunoprecipitation eluates were used to perform activity assays using the FRET assay (graph; 35 μl) and for western blots (WB; 10 μl). Signals obtained from non-immune IPs were subtracted from the A3G IPs for each sample. Western blots of 5% total immunoprecipitation input (T) is included for reference. Numbers on/above bars indicate actual values graphed. (C) Crude lysates from resting and activated primary CD4+ T cells were subjected to velocity sedimentation without RNase treatment, fractionated (1–12), and analyzed by western blotting with antibody to A3G. Bars indicate migration of HMM and LMM forms of A3G. All panels, except (C), are representative of at least three independent experiments. All FRET assays were performed with RNase A.

Finally, we examined the deaminase activity of endogenous A3G in primary human T cells. RNase A–treated cell lysates from both resting and activated CD4+ primary cells purified from peripheral blood exhibited much lower levels of deaminase activity than input cell lysates from 293FT cells containing equivalent amounts of A3G (Figure 5A, compare immunoblots of resting and activated CD4+ T cells to 293FT cells transfected with 10 ng and 30 ng A3G, respectively). Similar results were obtained when resting and activated CD4+ T cells were not treated with RNase A (unpublished data). Additionally, upon immunoprecipitation and RNase A treatment, minimal deaminase activity was detected in resting and activated CD4+ T cells, respectively, compared to similar amounts of immunoprecipitated A3G from 293FT cells (Figure 5B). Thus, the deaminase activity of endogenous A3G present in unstimulated and stimulated primary human CD4+ T cells was also inhibited relative to the deaminase activity of A3G transfected into epithelial cell lines, as was observed above for endogenous A3G in four human T cell lines. This was particularly surprising given that we and others [5] have observed that A3G in resting T cells is in the LMM form even in the absence of RNase treatment (Figure 5C). Thus, in T cell lines and in primary T cells, activity of LMM A3G is much lower than expected, and this low activity is most likely not attributable to inhibitory RNA since the low activity is observed even after RNase A treatment.

**Figure 6:**
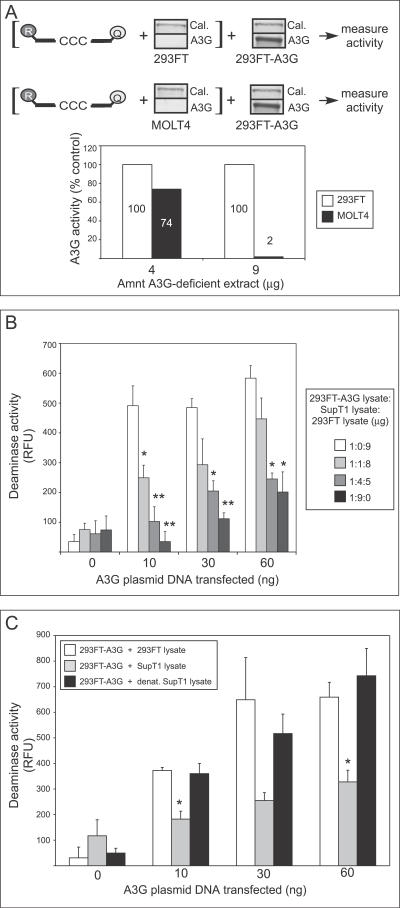
T Cell Lysates Inhibit Deaminase Activity of Exogenously Expressed A3G in 293FT Cell Lysates (A) Diagram explains experimental design. Lysate containing 4 or 9 μg of total cellular protein from non-transfected 293FT or MOLT4 cells was pre-incubated for 15 min with the dCdC- containing oligonucleotide (labeled with reporter [R] and quencher [Q] fluorophores), followed by the addition of lysate containing 1.0 μg of cellular protein from 293FT cells transfected with 1.0 μg of A3G plasmid DNA. Western blots show the amount of A3G protein in each lysate along with calreticulin (Cal.) as a loading control. A3G deaminase activity was then determined using the FRET-based assay. Activity measurements are graphed as percent of the activity of the 293FT control (to which non-transfected 293FT lysate was added). Numbers on/above bars indicate actual values graphed. (B) Lysate containing 1.0 μg of total cellular protein from 293FT cells transfected with the indicated amounts of A3G plasmid DNA was combined with the indicated amounts of lysate from A3G-deficient Sup T1 cells (representing 0, 1, 4, or 9 μg cellular protein). Total cellular protein in each sample was adjusted to 10 μg by adding lysate of non-transfected, A3G-deficient 293FT cells. Total cellular protein amounts (μg) for each of the three cell lysates separated by colons are indicated in box. A3G deaminase activity was then determined using the FRET assay and is graphed. Statistical significance relative to the A3G-deficient 293FT control (i.e., the 1:0:9 point), * *p* < 0.01, ** *p* < 0.001. Error bars represent standard error of the mean of triplicate reactions. (C) Lysate containing 1.0 μg of total cellular protein from 293FT cells transfected with the indicated amounts of A3G plasmid was combined with lysate containing 9 μg of total cellular protein derived from either non-transfected 293FT cells (open bars), SupT1 cells (grey bars), or SupT1 cells that were lysed and heated at 60 °C for 30 min (black bars). A3G deaminase activity was then determined using the FRET-based assay and is graphed. Statistical significance relative to 293FT control, * *p* < 0.01. Error bars represent standard error of the mean of triplicate reactions. All FRET assays were performed with RNase A. Background fluorescence obtained using the 13-mer GGG-containing oligonucleotide was subtracted from signal detected using the 13-mer CCC-containing substrate.

The low specific activity of A3G in T cells suggested three possible models: (1) T cells contain a less active isoform of A3G, (2) epithelial cell lines contain an activator of A3G that is absent or at lower levels in T cells, or (3) an inhibitor of A3G deaminase activity is present in T cells but not in epithelial cell lines (or is present in T cells at significantly higher levels). When we transfected 293FT cells with plasmids representing human A3G sequence variants that differ at two amino acid positions, one derived from A3G cDNA amplified from a CEM T cell line ([34]) and the other from A3G cDNA amplified from human kidney cells ([10]), we obtained similar deaminase-specific activity values (unpublished data), which argues against an inactive isoform present in T cells. To further distinguish among these possibilities, we first mixed lysate from non-transfected 293FT cells with lysate from H9 cells expressing endogenous A3G. Endogenous A3G activity from H9 cells was unchanged (unpublished data), suggesting that the activity observed in 293FT cells is not due to the presence of an activating factor. We next examined whether inhibition could be conferred to A3G-expressing 293FT lysates by simply adding lysates of human T cell lines. To keep A3G levels constant in these mixing experiments, we only added lysate from T cell lines (or control epithelial cell lines) that lacked detectable amounts of A3G. All lysates were RNase-treated in this experiment. First, we pre-incubated lysate from the A3G-deficient MOLT4 human T cell line or mock-transfected 293FT cells with labeled oligo, and then added cell lysate from 293FT cells expressing A3G (Figure 6A). Addition of a 4:1 ratio of crude MOLT4 lysate to A3G-expressing lysate led to a 26% reduction in activity relative to 293FT, while a 9:1 ratio reduced activity by 98% (Figure 6A, graph). Immunoblots of calreticulin showed similar levels of this cellular protein in 293FT and MOLT4 lysates (Figure 6A, blots). These data suggested the presence of an inhibitor in the MOLT4 cell line that can alter the activity of A3G expressed by transfection of epithelial cell lines. Furthermore, this inhibitor is not likely to be DNA or RNA, because inhibition was not altered by DNase (unpublished data) or by the RNase present in the assay.

We also examined whether the inhibitor is present in an additional A3G-deficient T cell line (SupT1). We altered our protocol so that A3G-expressing and A3G-deficient lysates were incubated together prior to the addition of oligo, and we examined lower amounts of A3G plasmid transfected into 293FT cells to more closely approximate physiological conditions. Addition of SupT1 cell lysate, but not lysate from non-transfected epithelial cell lines, produced a dose-dependent decrease in activity of transfected A3G at all levels of expression (Figure 6B). Notably, when A3G was expressed at approximately physiological levels (Figure 6B, 10 ng of A3G plasmid) in 293FT cells, a 1:1 ratio of crude T cell lysate to crude A3G-expressing 293FT lysate was sufficient to inhibit the deaminase activity of transfected A3G by 50%, and a 4:1 ratio reduced transfected A3G activity to background levels. As expected, proportionately less inhibition was seen when lysate was added to 293FT cell lysate expressing A3G at higher than physiologic levels (Figure 6B, 30 ng and 60 ng of A3G plasmid; also Figure 6A). Note that in this experiment, transfection efficiency was high, resulting in near saturating levels of A3G activity at 10 ng of transfected plasmid. Despite saturating levels of A3G activity, as A3G protein expression was increased with larger amounts of transfection plasmid, equivalent amounts of inhibitor became less effective. These data suggest that the inhibitor is present in limited quantities in SupT1 cells, allowing it to be titrated out by high levels of A3G. Moreover, although the inhibitory activity in T cell lysates was unaffected by DNase (unpublished data) or RNase, it was abolished when SupT1 lysate was heat treated (Figure 6C). Together, these data suggest that a protein(s) that is expressed in multiple T cell lines but not in epithelial-derived cell lines is responsible for the observed inhibition.

**Figure 7:**
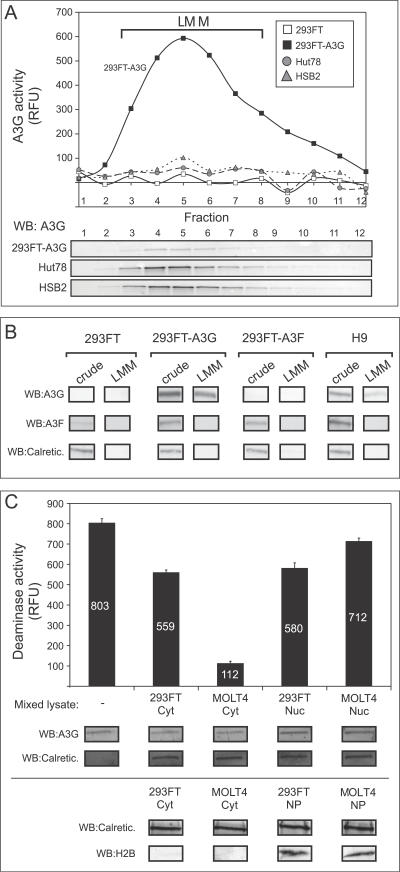
Active and Inhibited A3G Can Be Purified by Velocity Sedimentation (A) HMM A3G fractions from Hut78, HSB2, 293FT cells, or 293FT cells transfected with A3G plasmid DNA (293FT-A3G) were isolated, treated with RNase A, subjected to velocity sedimentation, and fractionated (1–12). Each fraction was assayed by both the FRET-based deaminase assay (graph) and by western blotting (WB). Migration of LMM A3G is indicated by brackets. (B) HMM A3G from 293FT cells, 293FT cells transfected with A3G plasmid DNA, 293FT cells transfected with A3F plasmid DNA, and H9 cells were treated with RNase and subjected to velocity sedimentation and fractionated. Aliquots of crude cell lysates and pooled gradient fractions 3–8 (LMM) were analyzed by western blot (WB) with antiserum specific to A3G, A3F, or calreticulin (Calretic.), and are shown below. (C) Cytoplasmic (Cyt) and nuclear (Nuc) extracts were prepared from either 293FT or MOLT4 T cells. Five μg of total protein from each extract, or water (-), was mixed with aliquots of pooled gradient fractions 3–8 from A3G-transfected 293FT cells containing 6 μg A3G (A and B), and A3G deaminase activity was determined using the FRET assay and was graphed. Below, western blots (WB) performed in parallel show the amount of A3G and the cellular control calreticulin (Calretic.) in each reaction. Note that the small amount of inhibition of deaminase activity seen upon addition of 293FT cytoplasmic extract and both nuclear extracts is most likely due to high levels of total protein input, which we previously observed to be mildly inhibitory (see Figure 3B). In an independent experiment (bottom), cytoplasmic extracts (Cyt) and nuclear pellets (NP) were analyzed for calreticulin (Calretic.) and the nuclear marker histone 2B (H2B). Error bars represent the standard error of the mean from triplicate reactions. Numbers on/above bars indicate actual values graphed. (A and C) All FRET assays were performed with RNase A. Background fluorescence obtained using GGG-containing oligonucleotides was subtracted from signal detected using the CCC-containing substrate.

To further confirm the existence of an inhibited form of A3G in T cell lines, we partially purified LMM A3G from T cell lines and transfected 293FT cells by velocity sedimentation. A3G protein was primarily recovered in the LMM fractions for all cell lines after RNase A treatment followed by velocity sedimentation (Figure 7A, blots). LMM A3G from transfected 293FT cells displayed high levels of deaminase activity, as expected. In contrast, even larger amounts of LMM A3G isolated from endogenously expressing HSB2 and Hut78 T cell lines displayed negligible deaminase activity (Figure 7A, graph). To rule out the possibility that A3F, another APOBEC expressed in non-transfected 293FT cells and T cell lines, was responsible for the deaminase activity, we used A3F-specific and A3G-specific antisera to demonstrate that although A3F is present in crude 293FT and H9 cell extracts, it does not co-migrate with A3G in LMM fractions (Figure 7B). This finding is consistent with previous observations that A3F-containing HMM complexes are more RNase resistant than A3G-containing HMM complexes [37]. Thus, these results, which were obtained using an independent method, further demonstrate the existence of an enzymatically inhibited form of A3G in T cells.

Finally, we investigated whether the A3G inhibitor resides in the cytoplasm or in the nucleus of T cells. Microscopic examination of crude cell lysates revealed that they are largely free of nuclei following clarification (unpublished data). Nevertheless, it is possible that small amounts of nuclear contents leak into the supernatant prior to this clarification step. To examine the subcellular localization of the inhibitor, we mixed aliquots of LMM gradient fractions prepared from 293FT cells expressing A3G with either cytoplasmic or nuclear extracts (containing 5 μg of total protein) from non-transfected 293FT cells or MOLT4 cells, a T cell line that does not express significant quantities of A3G (see Figure 3A). Addition of the MOLT4 cytoplasmic extract inhibited deaminase activity of A3G expressed exogenously in 293FT cells to a much greater extent than did cytoplasmic extract from non-transfected 293FT cells (Figure 7C), consistent with the results displayed in Figure 6. In contrast, when the same amount of total protein was added in the form of nuclear extract from either MOLT4 or 293FT cells, little inhibition of A3G activity was observed (Figure 7C). These data suggest that T cell cytoplasm, rather than T cell nuclei, is the primary source of the inhibitory activity. Thus, it is unlikely that leakage of nuclear contents during harvest accounts for inhibition of A3G deaminase activity in T cells.

## Discussion

In this study, we used two different cytidine deaminase assays—a previously described gel-based assay and a novel FRET-based assay—to compare A3G enzymatic activity in both exogenously and endogenously expressing cells. Although previous studies have documented deaminase activity in several cell types, including T cell lines and primary peripheral blood mononuclear cells (PBMCs) [30,31], the present study constitutes the first report to our knowledge in which deaminase activity of A3G, expressed endogenously or by transfection and normalized to the amount of A3G protein, is directly compared in a variety of different cell types. Surprisingly, we found that for comparable amounts of A3G protein, enzymatic activity is much lower in T cell lines (Figures 2–4) and in resting or activated primary T cells (Figure 5) than it is in epithelial-derived cell lines. These findings are consistent with a model in which (1) T cells contain an inhibitor of A3G activity, (2) epithelial-derived cell lines contain an activator of A3G activity, or (3) T cells contain a less active isoform of A3G. In support of the model in which an A3G inhibitor is expressed in T cells, we demonstrated that in the presence of RNase A or DNase, addition of lysate from A3G-deficient T cell lines (SupT1 or MOLT4) inhibits activity of A3G expressed in epithelial cells (Figures 6 and 7C), whereas addition of lysate from epithelial-derived cell lines did not activate A3G from T cells. The ability of T cell lysates to confer inhibition to A3G expressed exogenously in 293FT cells also argues against an alternate isoform of A3G being present in T cells. Thus, these data suggest that when A3G is in the LMM form, i.e., in resting T cells or following conversion by RNase treatment [5], it is capable of being inhibited by an RNase-insensitive factor(s) that is present or enriched in human T cells but not in epithelial-derived cell lines.

Experiments examining deaminase activity, taken together with experiments examining inhibition of deaminase activity by T cell lysates, suggest that the inhibitor is active in at least six T cell lines (H9, CEM, HSB2, Hut78, MOLT4, and SupT1), as well as in resting and activated peripheral blood CD4+ T cells. Moreover, our studies suggest that the inhibitor most likely resides in the cytoplasm of T cells, which is where the vast majority of A3G is localized [38], rather than in the nucleus (Figure 7D), although additional studies will be needed to verify this. Furthermore, these studies suggest that the inhibitor is either absent, present in lower abundance, or much less active in the transformed epithelial cell lines that we examined (293FT and HeLa). Whether the inhibitory factor is present in macrophages or other non-hematopoietic cell lines remains to be determined. Moreover, its exact mechanism of action is not known, although our studies indicate that it is insensitive to RNase A and DNase, but is sensitive to heat. These data suggest that the inhibitor is either a protein that interacts with A3G or an enzyme that modifies A3G post-translationally. Recent studies have found numerous proteins associated with A3G, although the majority interact with it in an RNA-dependent manner [23,39,40] and are therefore unlikely to constitute the inhibitor described here. Similarly, other studies found that A3G associates with proteins that reside in P bodies in T cell lines and primary T cells, but these associations were also RNA dependent [40,41].

Although it is known that the deaminase activity of exogenously expressed A3G is inhibited when A3G associates with RNA in HMM complexes [5,20] and that large ribonucleoprotein complexes are present in T cells [21], other mechanisms for regulating A3G activity could also exist, especially given that A3G is in the LMM form in resting T cells. In fact, multiple mechanisms of post-translational regulation have been described for proteins related to A3G, including APOBEC1 and activation-induced deaminase (AID) [42–47]*.* Thus, the host cell likely has multiple mechanisms for regulating A3G, especially given that APOBEC family members with unchecked deaminase activity can be mutagenic, causing tumors in vivo (reviewed in [48]). While some immunofluorescence studies have demonstrated an exclusively cytoplasmic location for A3G in epithelial cell lines (offering one potential mechanism to protect the host genome) [38,49], another study reported that small amounts of A3G are present in nuclear fractions isolated from the H9 T cell lines [9], suggesting that A3G may undergo nuclear shuttling in response to specific stimuli or by a novel mechanism. Since A3A, A3B, A3C, and A3H are known to localize to the nucleus [49], it is clear that other mechanisms besides cytoplasmic localization are used to protect the host genome from A3 proteins. Moreover, expression of A3G in yeast resulted in mutation of the yeast genome [50]. This indicates that A3G can enter the nucleus in eukaryotic cells, and suggests that mechanisms for tightly regulating A3G deaminase activity are likely to exist [51]. Thus, the RNase-insensitive inhibition of A3G deaminase activity reported here is not completely surprising. Moreover, analogous forms of regulation, acting on other APOBEC3 family members, could exist. Little is known about the regulation of the related proteins A3F, which also has anti-retroviral activity [52], and A3A, A3B, and A3C, which act against endogenous retroelements [53–56]. Use of the FRET-based, high-throughput assay described here with modifications in the substrate sequence should permit assessment of whether other APOBEC3 proteins also exhibit reduced enzymatic activity when expressed endogenously.

Notably, the presence of a factor that inhibits activity of A3G in T cells is consistent with data from other groups suggesting that deamination by A3G may not be required in order for A3G to be active [5,16–19]. Others have shown that partial antiviral activity exists even when A3G enzymatic activity is mutationally inactivated [16–19]. Our data, taken together with studies of others [5,57], raise the possibility that enzymatic activity of wild-type A3G expressed in vivo may be inactivated by a naturally occurring inhibitor that reduces hypermutation but does not eliminate antiviral action. Although a detailed understanding of the effects of such an inhibitor must await its identification, such a mechanism for modulating A3G-mediated deamination could have multiple implications for our understanding of HIV pathogenesis. Since A3G can exert anti-HIV effects both when it is packaged into virions by producer cells and when it is present in resting T cells that are targets of infection, the role for an inhibitor may be different in these two contexts. The implications of an A3G inhibitor that is active in resting T cells is more obvious. The unexpected observation that HIV reverse transcripts isolated from resting T cells contain fewer mutations than expected from studies of A3G activity in epithelial cell lines [5] could be explained by the presence of a cellular inhibitor that blocks the A3G deaminase activity of LMM A3G, but does not inhibit antiviral activity that results from other mechanisms of A3G action.

On the other hand, a role for an inhibitor is less clear when A3G is packaged into virions. In the absence of Vif, HIV cannot initiate a productive infection in T cells. Reverse transcripts from ΔVif virons produced by H9 T cells show evidence of hypermutation [58], suggesting that the inhibitory factor must be removed or inactivated upon packaging of A3G into the virion or following initiation of reverse transcription. However, given that A3G that is packaged into virions can exert a deaminase-independent inhibitory effect on HIV replication [17,18], it is possible that some packaged A3G remains associated with the inhibitory factor and partially reduces A3G enzymatic activity. Further studies will be necessary to distinguish between these possibilities.

It is not yet known if and when this inhibitor acts in vivo. Numerous studies have detected G-to-A hypermutation with varying frequency in sequences from infected patient PBMCs [59,60–63]. A single study examined the percentage of potential A3F and A3G target sequences (**G**A and **G**G on the coding strand, respectively) that were altered within 70 hypermutated sequences and identified sequences containing from 20% to 94% G-to-A substitution at these motifs [60]. Hypermutation in patient samples could result from the generation of viral variants that lack Vif and consequently package A3G into virions [64], or, alternatively, from Vif-independent A3G activity in resting T cells [5]. Notably, striking variability in the amount of hypermutation has been observed both in samples from infected patients and in T cells infected with Vif-encoding wild-type virus in vitro [20,60]. In one study, the level of G-to-A hypermutation observed upon infection of CD4+ T cells with wild-type virus in vitro depended on the timing of infection relative to T cell activation [60], and therefore cannot be easily explained by sporadic generation of viral variants encoding mutations in Vif. In contrast, the RNase-independent inhibitor of A3G deaminase activity in T cells described here could account for at least some of the variability in G-to-A hypermutation observed upon infection of resting T cells, both in vitro and in patient samples. One implication of our findings is that such physiologic regulation of deaminase activity may need to be taken into account in studies correlating A3G mRNA and protein expression to pathogenesis. For example, variation in regulation of A3G deaminase activity in T cells could have contributed to conflicting results of studies addressing the consequences of A3G expression levels and polymorphisms for HIV disease susceptibility and progression [65–68].

Finally, it is possible that even modest amounts of inhibition of A3G deaminase activity in primary cells could have implications for the outcome of HIV infection in vivo. Others have suggested that sporadic inactivating mutations in Vif may generate a reservoir of diverse sequences that may function as a “genetic resource” to facilitate viral evolution [12,64]. While high levels of A3G activity would be expected to result in complete failure of virus propagation with antiviral consequences, reduced levels of A3G activity could result in virus that is viable and in fact exhibits increased rates of viral evolution due to the modest increase in mutation. Further study will be required to determine if the inhibitor described here can reduce the activity of endogenous A3G so that it fails to produce lethal hypermutation, and instead produces sub-lethal levels of mutation that benefit the virus by promoting viral adaptation. If so, then strategies that disrupt the inhibitor could help to increase T cell intrinsic immunity to HIV infection.

## Materials and Methods

### Plasmids.

All A3G plasmids transfected in this study were untagged. For exogenous expression of A3G, a construct obtained from National Institutes of Health (NIH) AIDS Research and Reference Reagent Program (catalog #9904, https://www.aidsreagent.org/; [10]) was modified to include Kozak's consensus sequence and to remove the MycHis tag. Data shown here were also confirmed using an A3G plasmid that was modified to correspond to the A3G cDNA sequence variant obtained from T cells [34], by using site-directed mutagenesis to mutate A1448 to G and T2071 to G. Constructs containing point mutations in the first and second A3G deaminase domains were obtained from Michael Malim (King's College, London, United Kingdom) [17] and modified to include the Kozak's sequence. All coding regions were confirmed by sequencing.

### Cell culture, transfection, and harvest.

Human 293FT (Invitrogen, http://www.invitrogen.com/) or 293T (ATCC, http://www.atcc.org/) cells were maintained in DMEM supplemented with 10% FBS and 1% pen-strep (Gibco, http://www.invitrogen.com/). Transfections were typically performed by mixing the indicated amounts of plasmid DNA and 15 μl of Lipofectamine 2000 (Invitrogen) in OPTIMEM (Gibco) and adding to cells plated in 60-mm dishes in antibiotic-free DMEM. Alternatively, for gradient purification experiments, DNA was mixed with 45 μl of Lipofectamine 2000 and added to cells plated in 100-mm dishes. For harvests, cells were washed 1× in cold PBS and collected 40–48 h post-transfection by scraping in 250 μl of NP40 buffer (0.626% NP40, 10 mM Tris acetate [pH 7.4], 50 mM potassium acetate, 100 mM NaCl) supplemented with protease inhibitor cocktail for mammalian cells (Sigma, http://www.sigmaaldrich.com/) with or without 10 mM EDTA, as previously described [35]. Cells were lysed by 20 passages through a 20-gauge catheter. Lysates were clarified by centrifugation at 1,000 rpm (162*g*) for 10 min in a GH 3.8 rotor using an Allegra 6R centrifuge (Beckman Coulter, http://www.beckmancoulter.com/). Supernatants and pellets were examined by light microscopy to confirm that nuclei were removed by clarification. Supernatants were then subjected to a 30-s spin in a microfuge at 18,000*g*. The supernatants were removed again and used as crude cell lysates. The H9, SupTI, Jurkat, MOLT4, CEM (ATCC), and CEM-SS and C8166 (NIH AIDS Research and Reference Reagent Program) human T cell lines, and the U937 monocyte cell line (ATCC), were maintained in RPMI supplemented with 10% FBS and 1% pen-strep (Invitrogen). Hut78 and HSB2 cells (ATCC) were maintained in IMDM (Invitrogen) supplemented with 1% pen-strep and 20% and 10% FBS, respectively. To prepare cell lysates, intact cells were pelleted at 1,000 rpm (162*g*) × 10 min in a Beckman Allegra 6R using a GH-3.8 rotor, washed 1× in cold PBS, and lysed in 250 μl lysis buffer per 1.5 × 10^7^ cells as described for 293FT cells. Generation of H9 cells stably expressing HIV-1 encoding a deletion in envelope from bp 6636 to 7216, a frameshift in Vpr created by addition of four nucleotides at the unique NcoI site at bp 5260, a deletion in Vif between bp 4707 and 4989, and a deletion of Nef from bp 8390 to 8661, was previously described [69].

### Primary CD4+ T cell stimulation.

Human blood samples were obtained after informed consent in accordance with procedures approved by the human ethics committee of the Benaroya Research Institute. PBMCs were prepared by centrifugation over Ficoll-Hypaque gradients. CD4+ T cells were purified by depletion of cells expressing CD8, CD14, CD16, CD19, CD36, CD56, CD123, TCRγ/δ, and CD235a with the CD4+ No-touch T cell isolation kit (Miltenyi Biotec, http://www.miltenyibiotec.com/). Cells were cultured in RPMI supplemented with 10% pooled human serum only or were activated in culture by the addition of 500 ng/ml PMA (Sigma) and 325 IU/ml IL-2 (Chiron, http://www.chiron.com/). Cells were harvested after 72 h for activity measurements. For proliferation assays, cells were cultured in 96-well plates (30,000–50,000/well) for 72 h at 37 °C with 1 μCi [^3^H]thymidine added for the last 20 h of incubation. Cells were harvested and ^3^H uptake was measured on a 1450 Microbeta Plus liquid scintillation counter (Wallac, http://las.perkinelmer.com/).

### FRET-based activity assay from cell lysate.

Quantification of total protein in cell lysates was performed using the BCA protein assay kit (Pierce, http://www.piercenet.com/) according to the manufacturer's protocol. For each sample, activity was measured in triplicate for 2-fold serial dilutions ranging from 0.078 μg to 20 μg of total protein. Typically, four to eight dilutions were assayed for each condition. Assays were performed in 96-well iQ PCR plates (Bio-Rad, http://www.bio-rad.com/) using 6-FAM- and TAMRA-labeled custom Taqman probes (Applied Biosystems, http://www.appliedbiosystems.com/). Sequences are shown in Table S1. To each well was added 10 μl of cell lysate in NP40 buffer and 70 μl of a master mix containing 10 pmol Taqman probe, 0.4 units uracil DNA glycosylase (NEB, http://www.neb.com/), 50 mM Tris (pH 7.4), and 10 mM EDTA. For RNase treatment, RNase A (Qiagen, http://www.qiagen.com/) was added to the master mix to a final concentration of 250 μg/ml. For DNase treatment, lysates were prepared in EDTA-free lysis buffer to which 10x start buffer and RQ RNase-free DNase (0.5 U/100 ul; Promega, http://www.promega.com/) were added. Lysates were incubated at 37 °C for 15 min and then the DNase was inactivated by addition of 10x stop buffer. The plates were incubated at 37 °C for 1.5–2 h followed by addition of 4 μl of 4N NaOH and incubation for 30 min at 37 °C. The wells were then neutralized by additional of 4 μl of 4N HCl and 36 μl of 2 M Tris (pH 7.9). The plates were cooled to 4 °C and fluorescence was measured using an iCycler (Bio-Rad) using the 490 nM FAM setting. Standard cleavage curves were prepared by mixing different ratios of dual-labeled probe with two singly labeled half probes to generate a linear, highly reproducible 10-fold increase in signal over the range of 0% to 100% cleavage (Figure S1). For Figures 2A and 3–7, background fluorescence obtained for each sample using GGG-containing oligonucleotides was subtracted from the signal detected using the CCC-containing substrate. Alternatively, in Figures 2B and S3, the activity present in non-transfected HeLa cells using a CCC-containing oligo was subtracted from all samples. Protein expression was analyzed by SDS-PAGE followed by transfer to a nitrocellulose membrane (GE Osmonics, http://www.osmolabstore.com/). Western blotting was performed with antisera specific for human A3G [17] and a monoclonal antibody to calreticulin (Affinity Bioreagents, http://www.bioreagents.com/), a loading control protein, followed by detection with alkaline phosphatase-conjugated anti-rabbit IgG and incubation with Western Blue substrate. A3F antiserum used for western blotting was a gift of Michael Malim. Antisera to histone 2B (Upstate, catalog number 07–371, http://www.upstate.com/) was used as a nuclear marker.

### Gel-based activity assay from cell lysate.

For each sample, 5 μl of cell lysate containing 10 μg of total cellular protein was added to 20 μl of assay buffer containing 50 mM Tris, 10 mM EDTA, and 0.1 pmol IR700-labeled oligonucleotide at pH 7.4 and incubated at 37 °C for 2 h. Reactions were stopped by addition of 15 μl of stop buffer and heating to 95 °C as previously described [24]. Samples were then electrophoresed on 15% TBE-urea polyacrylamide gels (Bio-Rad). The IR700 infrared tag was detected by a LI-COR infrared scanner (LI-COR Biosciences, http://www.licor.com/) and quantitated by ImageJ software.

### Immunoprecipitations.

Clarified cell lysates (containing 300–500 μg of total protein) were subjected to immunoprecipitation with an antiserum to A3G (generated against the C-terminal 29 amino acids of human A3G and described previously [17] that was affinity purified against the A3G peptide or rabbit IgG coupled to protein A immobilized on Tris Acryl beads (Pierce) as described previously [35,70]. After washing, all buffer was removed by aspiration. For assay of eluted A3G, the beads were incubated for 1 h in a 37 °C shaker with 80 μl of elution buffer containing 50 mM Tris, 10 mM EDTA (pH 7.4), and RNase A at 250 μg/ml, after which eluates were removed from beads. Variable amounts of eluate were mixed with fresh elution buffer to a final volume of 70 μl. This was then added to 10 μl of master mix containing 10 pmol Taqman probe, 0.4 units uracil DNA glycosylase, 50 mM Tris (pH 7.4), and 10 mM EDTA, and assayed as described for cell lysates. Alternatively, immunoprecipitations (IPs) were performed in the presence of RNase A. Following washings, beads were incubated for 1–2 h in a 37 °C shaker with 80 μl of activity assay master mix containing 10 pmol Taqman probe, 0.4 units uracil DNA glycosylase, 50 mM Tris (pH 7.4), and 10 mM EDTA. The master mix was removed and assayed as described for cell lysates.

### Velocity sedimentation.

For analysis of A3G-containing complexes (primary T cells), 80–100 μl of cell lysates were layered onto step gradients containing 900 μl each of 10%, 15%, 20%, 30% and 50% sucrose in NP40 buffer with EDTA and subjected to velocity sedimentation in a Beckman MLS 50 rotor at 45,000 rpm (163000*g*) for 45 min at 4 °C. Fractions (200 μl) were serially collected from the top of the gradient, precipitated with trichloroacetic acid, and analyzed by immunoblotting. Alternatively, 80–100 μl of cell lysates (293FT and H9 cells) were layered onto 5-ml continuous gradients of 5%–25% sucrose in NP40 buffer with EDTA and subjected to velocity sedimentation in a MLS 50 rotor at 45,000 rpm for 90 min at 4 °C. Fractions (400 μl) were serially collected from the top of the gradient and aliquots analyzed by immunoblotting.

### Isolation of LMM fractions.

For 293FT cells, four 100-mm plates of cells were lysed in 3 ml of NP40 buffer supplemented with protease inhibitors and 10 mM EDTA, as described above. For T cells, 50 × 10^6^ cells were lysed in 3 ml of NP40 buffer supplemented with protease inhibitors and 10 mM EDTA. For isolation of HMM complexes, 3 ml of crude cell lysates were layered over 2 ml of 20% sucrose in NP40 buffer and subjected to velocity sedimentation in a Beckman MLS 50 rotor at 50,000 rpm (201,000*g*) for 80 min at 4 °C. The bottom 500 μl was collected and sucrose was removed using 2-ml Zeba Desalt Columns (Pierce) prewashed with NP40 buffer. The flow through was treated with RNase A (250 μg/ml) for 30 min at 26 °C, and precipitated material was removed with a 30-s spin in a microfuge at 18,000*g*. For isolation of LMM fractions, 400 μl of the RNase-treated material was layered onto 5-ml continuous gradients of 5%–25% sucrose in NP40 buffer and subjected to velocity sedimentation in a MLS 50 rotor at 50,000 rpm for 3.5 h at 4 °C. Fractions (200 μl) were serially collected from the top of the gradient and fractions 1–12 were analyzed by immunoblotting.

### Preparation of cytoplasmic and nuclear extracts.

For adherent cells, 10-cm confluent plates were washed 1× in cold PBS and then cells were collected by scraping in 1 ml of cold PBS. Cells were pelleted at 1,000 rpm (162*g*) × 10 min in a Beckman Allegra 6R using a GH-3.8 rotor and the PBS was removed. Cells were resuspended in 1 packed cell volume (PCV) of NE1 buffer (10 mM HEPES [pH 8.0], 1.5 mM MgCl_2_, 10 mM KCl, 1 mM DTT, 1:100 protease inhibitor cocktail) and incubated on ice for 15 min. Cells were then sheared by five passages through a 22-gauge needle and subjected to a 30-s spin in a microfuge at 18,000*g*. The supernatant was collected and used as “cytoplasmic extract.” The pellet was washed 1× in an additional 1 PCV PBS. The remaining pellet was resuspended in 2/3 PCV NE2 buffer (20 mM HEPES [pH 8.0], 1.5 mM MgCl_2_, 25% glycerol, 420 mM NaCl, 0.2 mM EDTA, 1 mM DTT, 1:100 protease inhibitor cocktail) and incubated at 4 °C with shaking for 30 min, followed by a 5-min spin in a microfuge at 18,000*g*. The supernatant was collected and used as “nuclear extract.” The pelleted material was designated “nuclear pellet.” For suspension cultures, cells were pelleted and washed 2× in cold PBS. Extracts were prepared as described for adherent cells.

### Lysate mixing experiments.

Lysates from A3G-transfected 293FT cells, non-transfected 293FT cells, and A3G-deficient T cell lines were prepared as previously described. In Figure 6A, lysates containing 4 or 9 μg of protein from either the MOLT4 T cell line or 293FT cells were pre-incubated with the FRET assay master mix containing labeled oligo, RNase, and UDG for 15 min at 37 °C. One μg protein from 293FT cells expressing A3G was added per well and the reactions were incubated at 37 °C for an additional 1.5 h. In Figure 6B, lysates containing 0, 1, 4, or 9 μg of protein from the SupT1 T cell line was mixed with 1 μg of protein from 293FT cells expressing A3G. Lysate from non-transfected 293FT cells was added to bring the total protein to 10 μg. Mixed lysates were incubated for 1 h at 37 °C and then FRET assay master mix was added. In Figure 6C, lysates from non-transfected 293FT or SupT1 cells were heated at 60 °C for 30 min or held on ice prior to mixing with lysates from transfected 293FT cells. Mixed lysates were incubated for 1 h at 37 °C and then FRET assay master mix was added. For Figure 7C, nuclear and cytoplasmic extracts were prepared as described above. For each condition, 5 μg of nuclear or cytoplasmic protein from 293FT or MOLT4 cells or water was mixed with 2 μl of pooled LMM fractions 3–8 prepared as described above for A3G-transfected 293FT cells. The mixed lysates were incubated for 1 h at 37 °C and then FRET assay master mixes containing either the 21-mer ACCCA or 21-mer GGG oligo were added.

### Quantitation of specific activity.

To calculate specific activity, deaminase activity was measured using different amounts of input (1.25 to 10 μg of total cellular protein) to generate dose-response curves for T cell lines and a comparable epithelial cell line control. Western blotting was used to determine the amount of A3G in 10 μg of total cellular protein (the highest point in the linear range) for each cell type. A standard curve was generated by western blotting different amounts of purified recombinant A3G in parallel with cell lysate samples. A3G bands on western blots were quantified by densitometry, and ng amounts of A3G were determined for each sample by interpolation on the standard curve of recombinant A3G (Figure S3). For each cell type, deaminase activity of the 10-μg sample was divided by A3G protein content of that sample to obtain the specific deaminase activity.

For densitometry, immunoblots were scanned using a Cannon 8400F scanner and quantitated relative to recombinant standards (produced by ImmunoDiagnostics, http://www.immunodx.com/, NIH AIDS Research and Reference Reagent Program catalog #10067) present on the same blot using Adobe Photoshop. Statistical analyses were performed using a Student's *t*-test (one tailed, assuming unequal variances).

## Supporting Information

Figure S1 A Quantitative, High-Throughput FRET-Based Assay for Measuring A3G Deaminase Activity (A) Schematic diagram depicting the FRET-based A3G activity assay. (1) In transfected 293T cells, A3G exists in an enzymatically inactive, HMM complex and is unable to deaminate the CCC-containing oligonucleotide substrate. The substrate remains uncleaved throughout subsequent steps, and consequently, the reporter fluorophore (R) is quenched by the quencher fluorophore (Q) and background fluorescence is detected. (2) In transfected 293T cells, RNase A treatment disrupts the HMM complex, converting A3G to an LMM, enzymatically active form. Upon incubation with substrate, deamination by A3G results in conversion of dC residues to dUs (a). Treatment with uracil DNA glycosylase (UDG) leads to removal of dUs to create abasic sites (b). At high pH, the substrate is cleaved at the site of deamination (c), dissociating the reporter and quencher fluorophores and creating a fluorescent signal that can be detected by a luminometer (d). Note that depending on the oligo used, other detection methods can be used in step (d) to measure deaminase activity, as in Figure 1. (B) Standard cleavage curves were prepared by mixing different ratios of dual-labeled oligonucleotides with two singly labeled half oligonucleotides (shown in Table S1 as 6-FAM half probe and TAMRA half probe) to simulate a range from 0% to 100% cleavage (% free reporter). Graph shows RFU measurements for each ratio of probes. Error bars represent standard error of the mean from triplicate samples.
(52 KB PDF)

Figure S2 Validation of the FRET-Based Assay by Measuring Exogenous Wild-Type and Mutant A3G Transfected into Epithelial Cell Lines (A) Activity measurements using the FRET-based assay were performed on lysates of 293FT cells transfected with increasing amounts of A3G plasmid DNA, as indicated. Assays were performed either with (+) or without (-) RNase A, using a 13-base oligonucleotide substrate either containing the A3G recognition sequence (CCC) or lacking dCs (GGG), as indicated. A3G deaminase activity is graphed for each condition. A3G expression was assessed by western blot (WB) of three amounts of total cellular protein input (10, 30, and 90 μg), with western blots of calreticulin (Calretic.) as a loading control. (B) A3G deaminase activity using the FRET-based assay was determined for increasing amounts (as indicated by amount of total cellular protein in μg) of RNase A–treated cell lysate obtained from 293FT cells transfected with 100 ng of A3G plasmid using oligonucleotide substrates (listed in Table S1) ranging in length from 13 to 19 bases or 13-base substrates lacking the A3G recognition sequence (GGG or GTC) as indicated. A3G deaminase activity is graphed, showing a dose response with increasing amounts of A3G lysate only when substrates containing the canonical A3G recognition sequence (CCC) were used. (C) Non-transfected 293FT cells, or 293FT transfected with either A3G and A3F plasmid DNA, was analyzed by western blot using antiserum specific for A3G, A3F, and the loading control calreticulin. (D) Lysates from 293FT cells expressing A3G were subjected to parallel immunoprecipitations using antiserum specific for A3G or a non-immune control. A3G was eluted in the presence of RNase A and A3G deaminase activity from increasing amounts of the eluates was determined using the FRET-based assay using a 13-base CCC-containing oligonucleotide. The fluorescent signal from a matched non-immune immunoprecipitation was subtracted from each A3G immunoprecipitation and is graphed. Graph shows a dose response obtained from increasing input amounts of A3G specific elution. (E) Deaminase activity of RNase A treated lysates from 293FT cells expressing either wild-type (WT) A3G or A3G constructs encoding point mutations in both A3G catalytic sites was determined using the FRET-based assay and was graphed. Background fluorescence obtained using a GGG-containing oligonucleotide was subtracted from each sample. Below, western blot analysis of equivalent amounts of lysates showing expression of A3G constructs and calreticulin, as a loading control. Similar results were observed following immunoprecipitation of these mutants (unpublished data). Statistical significance relative to WT, * *p* < 0.02, ** *p* < 0.03. Error bars represent standard error of the mean from triplicate reactions.
(206 KB PDF)

Figure S3 Example Calculation of Specific Deaminase Activity (A) A3G content of each sample was determined by subjecting the cell lysates as well as known amounts of recombinant A3G (ng rA3G) to western blotting with antibody to A3G. Densitometry of the rA3G bands was used to create a standard curve of A3G protein amounts and to determine the linear range of the western blot (graph). The amount of A3G for each sample (HeLa-A3G, H9, and CEM) was then determined by interpolation on this graph. (B) Deaminase activity was determined over a range of input amounts (1.25–10 μg total cellular protein) for RNase A–treated crude cell lysates from the T cell lines H9 and CEM as well as HeLa cells transfected with 3 μg of A3G plasmid (graph). Specific activity calculations for all samples were performed for the total protein amount (10 μg) corresponding to the high end of the linear range for activity of transfected HeLa cells. Background fluorescence obtained for each sample using a non-transfected HeLa control was subtracted from each sample to obtain the corrected deaminase value (indicated on graph for 10-μg inputs). Specific activity was calculated by dividing the corrected deaminase value in RFU by the amount of A3G protein (ng A3G). The specific activity for these samples are graphed in Figure 2B.
(218 KB PDF)

Table S1 Click here for additional data file. Oligonucleotide Probe Sequences
Target motifs are in bold.
(17 KB XLS)

## Abbreviations

A3G: APOBEC3G
dC: deoxycytidine
dU: deoxyuridine
FRET: fluorescence resonance energy transfer
HMM: high molecular mass
IP: immunoprecipitation
LMM: low molecular mass
RFU: relative fluorescence unit
UDG: uracil DNA glycosylase
Vif: viral infectivity factor
