# Supplementary material for: T Cells Contain an RNase-Insensitive Inhibitor of APOBEC3G Deaminase Activity
Source: PLoS Pathog. 2007 Sep 21;3(9):e135. doi: 10.1371/journal.ppat.0030135 (PMC1993843; doi:10.1371/journal.ppat.0030135)
Supplement: Supplementary file 1 [file ppat.0030135.sg001.pdf]

Figure S1

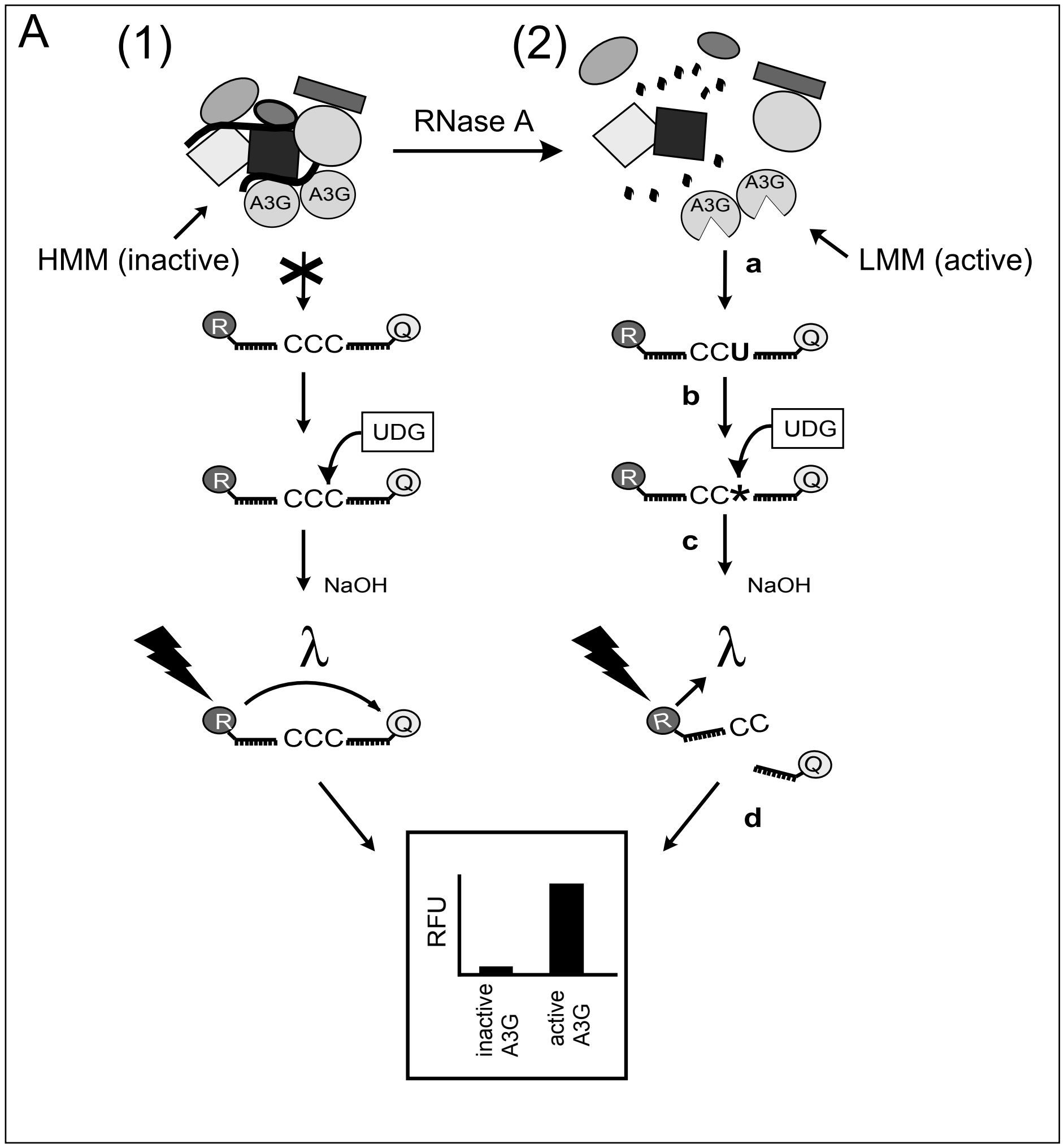

A
(1)
(2)
RNase A
A3G
A3G
HMM (inactive)
LMM (active)
R
CCC
Q
CCU
a
b
c
d
UDG
CC*
NaOH
λ
CC
RFU
inactive A3G
active A3G

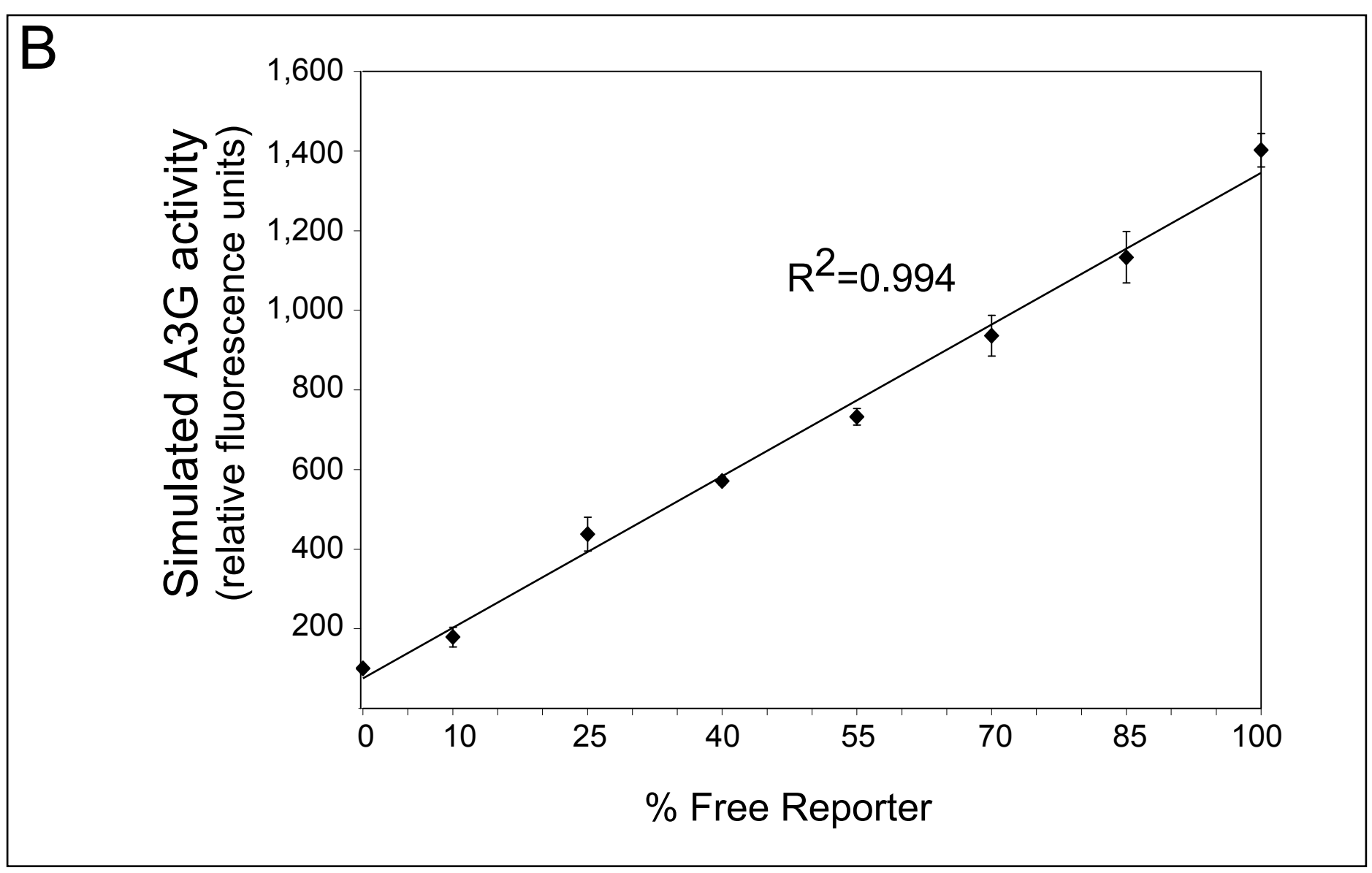

B
Simulated A3G activity
(relative fluorescence units)
1,600
1,400
1,200
1,000
800
600
400
200
R^2=0.994
0
10
25
40
55
70
85
100
% Free Reporter
