# Supplementary material for: T Cells Contain an RNase-Insensitive Inhibitor of APOBEC3G Deaminase Activity
Source: PLoS Pathog. 2007 Sep 21;3(9):e135. doi: 10.1371/journal.ppat.0030135 (PMC1993843; doi:10.1371/journal.ppat.0030135)
Supplement: Supplementary file 2 [file ppat.0030135.sg002.pdf]

Figure S2

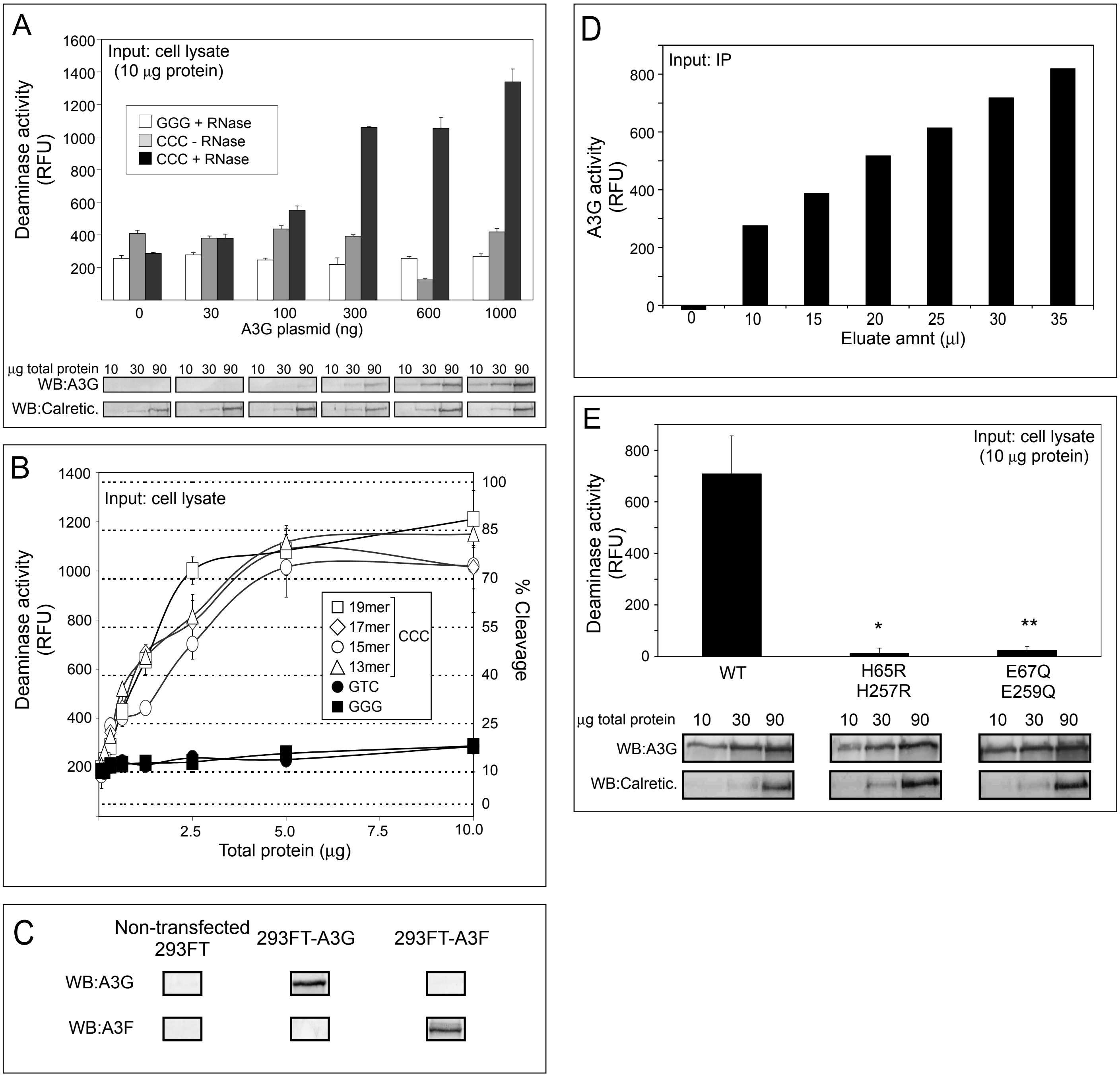

A
Input: cell lysate
(10 μg protein)
GGG + RNase
CCC - RNase
CCC + RNase
Deaminase activity
(RFU)
A3G plasmid (ng)
μg total protein 10 30 90
WB:A3G
WB:Calretic.
B
Input: cell lysate
19mer
17mer
15mer
13mer
CCC
GTC
GGG
Deaminase activity
(RFU)
% Cleavage
Total protein (μg)
C
Non-transfected
293FT
293FT-A3G
293FT-A3F
WB:A3G
WB:A3F
D
Input: IP
A3G activity
(RFU)
Eluate amnt (μl)
E
Input: cell lysate
(10 μg protein)
Deaminase activity
(RFU)
WT
H65R
H257R
E67Q
E259Q
μg total protein 10 30 90
WB:A3G
WB:Calretic.
