# Supplementary material for: T Cells Contain an RNase-Insensitive Inhibitor of APOBEC3G Deaminase Activity
Source: PLoS Pathog. 2007 Sep 21;3(9):e135. doi: 10.1371/journal.ppat.0030135 (PMC1993843; doi:10.1371/journal.ppat.0030135)
Supplement: Supplementary file 3 [file ppat.0030135.sg003.pdf]

Figure S3

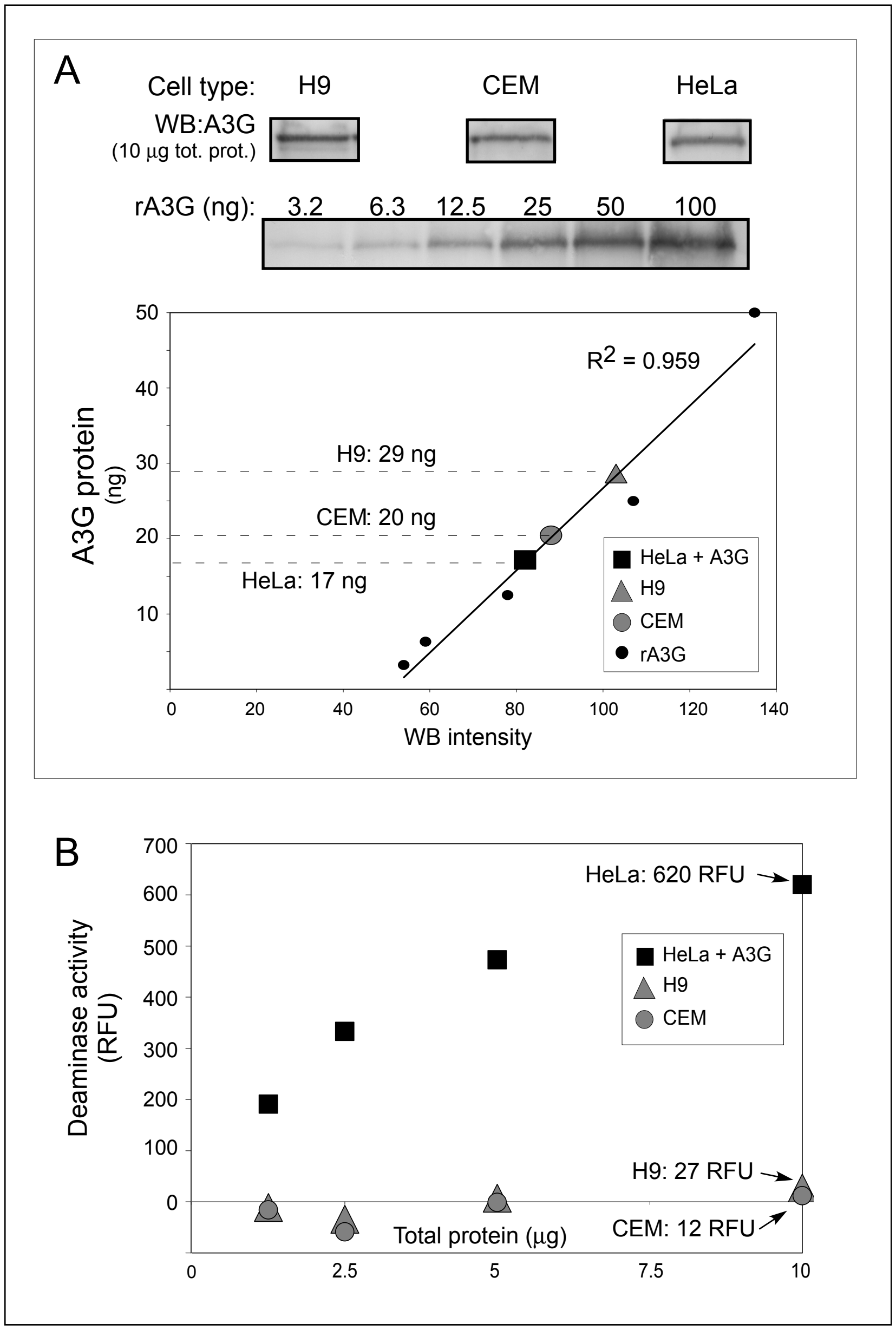
A
Cell type:
H9
CEM
HeLa
WB:A3G
(10 µg tot. prot.)
rA3G (ng):
3.2
6.3
12.5
25
50
100
R2 = 0.959
H9: 29 ng
CEM: 20 ng
HeLa: 17 ng
A3G protein
(ng)
WB intensity
HeLa + A3G
H9
CEM
rA3G
0
20
40
60
80
100
120
140
10
20
30
40
50
B
HeLa: 620 RFU
H9: 27 RFU
CEM: 12 RFU
Deaminase activity
(RFU)
Total protein (µg)
HeLa + A3G
H9
CEM
0
100
200
300
400
500
600
700
0
2.5
5
7.5
10
